# The impact of leukocyte‐associated RNA retention on platelet RNA‐based cancer detection

**DOI:** 10.1002/1878-0261.70353

**Published:** 2026-10-30

**Authors:** Michał Sieczczyński, Krzysztof Pastuszak, Anna Samelak‐Czajka, Zuzanna Cemka, Paulina Jackowiak, Tomasz Stefaniak, Sylwia Łapińska‐Szumczyk, Jacek Jassem, Anna J. Żaczek, Matthew T. Rondina, Myron G. Best, Sjors G. J. G. In 't Veld, Anna Supernat

**Affiliations:** ^1^ Laboratory of Translational Oncology Intercollegiate Faculty of Biotechnology of the University of Gdansk and the Medical University of Gdansk Poland; ^2^ Centre of Biostatistics and Bioinformatics Medical University of Gdansk Poland; ^3^ Department of Software Engineering, Faculty of Electronics, Telecommunications and Informatics Gdansk University of Technology Poland; ^4^ Department of Algorithms and Systems Modelling, Faculty of Electronics, Telecommunications and Informatics Gdansk University of Technology Poland; ^5^ Laboratory of Single Cell Analyses, Institute of Bioorganic Chemistry Polish Academy of Sciences Poznań Poland; ^6^ Department of Biomedical Engineering, Faculty of Electronics, Telecommunications and Informatics Gdansk University of Technology Poland; ^7^ Department of Quality in Healthcare Medical University of Gdansk Poland; ^8^ Board of Directors University Clinical Center, Medical University of Gdansk Poland; ^9^ Department of Oncologic, General and Transplant Surgery University Clinical Centre, Medical University of Gdansk Gdansk Poland; ^10^ Department of Obstetrics and Gynecology, Gynecological Oncology and Endocrinological Gynecology University Clinical Center Gdansk Poland; ^11^ Department of Oncology and Radiotherapy, Faculty of Medicine Medical University of Gdansk Gdansk Poland; ^12^ Molecular Medicine Program University of Utah Salt Lake City UT USA; ^13^ George E. Wahlen Veterans Affairs Medical Center, Department of Internal Medicine and the Geriatric Research Education and Clinical Center (GRECC) Salt Lake City UT USA; ^14^ Department of Pathology University of Utah Salt Lake City UT USA; ^15^ Division of General Internal Medicine, Department of Internal Medicine University of Utah Salt Lake City UT USA; ^16^ Department of Neurosurgery Amsterdam UMC Location Vrije Universiteit Amsterdam Amsterdam the Netherlands; ^17^ Cancer Center Amsterdam and Brain Tumor Center Amsterdam Amsterdam the Netherlands; ^18^ Cancer Center Amsterdam Amsterdam UMC The Netherlands; ^19^ Amsterdam Public Health, Amsterdam UMC, Vrije Universiteit University of Amsterdam Amsterdam The Netherlands; ^20^ Translational AI in Laboratory Medicine, Department of Laboratory Medicine Amsterdam UMC Amsterdam The Netherlands

**Keywords:** cancer detection, leukocyte‐associated RNA retention, liquid biopsy, machine learning, RNA sequencing, tumor‐educated platelets

## Abstract

Platelet RNA profiling is a promising liquid biopsy approach, offering minimally invasive insights into systemic diseases, such as cancer. Yet, the effect of leukocyte‐associated RNA retention on platelet transcriptomic signatures remains unclear. This study provides the first comprehensive evaluation of how leukocyte‐associated RNA retention influences platelet RNA sequencing and machine learning (ML)‐based cancer detection. Using the largest publicly available platelet transcriptomic dataset (2351 human RNA samples) and single‐cell/single‐platelet RNA sequencing from 29 donors, we quantified leukocyte‐associated RNA retention in matched sample groups (low vs. high retention) via a novel 132‐gene leukocyte marker panel. Diagnostic implications were assessed using 10 ML algorithms for binary classification (cancer vs. healthy), examining the contribution of leukocyte‐derived transcripts. Interestingly, models trained on high‐retention samples often showed slightly improved performance, suggesting complementary diagnostic value from leukocyte signals. Importantly, *post hoc* correction using remove unwanted variation (RUV) and linear modeling effectively mitigated retention effects. However, adding the leukocyte‐associated RNA retention score to the full‐cohort classifier changed cancer‐detection AUC only minimally (0.9247 to 0.9249). Thus, whether this signal should be corrected, recalibrated, or reported should depend on the study question rather than assuming routine removal.

## Graphical abstract

Platelets carry RNA signals that may help detect cancer, but platelet samples can also retain RNA from white blood cells. We found that this retained white blood cell signal did not significantly affect machine learning cancer classification, suggesting that the cancer‐related RNA signal in platelets is largely independent of leukocyte‐associated RNA. Graphical abstract created using OpenAI image generation in ChatGPT.

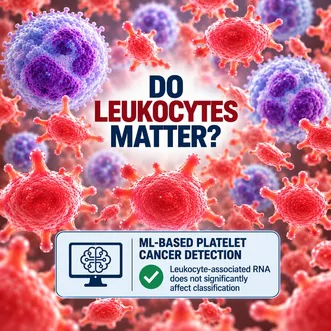

**Abbreviations**
AUC ROC: area under the receiver operating characteristic curve
CPM: counts per million
FDR: false discovery rate
KNN: k‐nearest neighbors
ML: machine learning
PBMCs: peripheral blood mononuclear cells
PCA: principal component analysis
QC: quality control
RUV: remove unwanted variation
SVM: support vector machine
TEPs: tumor‐educated platelets
UMAP: Uniform Manifold Approximation and Projection
UMI: unique molecular identifier

## 1 Introduction

Liquid biopsies have emerged as a valuable source of information, complementing traditional tissue biopsies and imaging techniques, and attracting substantial attention from molecular biologists, clinicians, and patients. This minimally invasive approach enables the detection, characterization, and monitoring of various diseases in a convenient, repeatable, and real‐time manner [1, 2, 3]. Among the components analyzed in liquid biopsies, platelets stand out due to their ability to dynamically mirror host responses to pathological processes, therapeutic interventions, and physiological changes [4, 5].

Platelets play essential roles in a wide range of physiological processes, including maintaining homeostasis, immune response, angiogenesis, and promoting wound healing. Their transcriptomic landscape has been extensively characterized in both health and disease contexts [6, 7, 8, 9, 10]. It is estimated that each platelet contains approximately 20 million protein molecules, representing around 5000 macromolecular species [11]. Some of these proteins originate from mRNAs transcribed and translated in megakaryocytes during thrombopoiesis, while others are synthesized *de novo* in circulating platelets. Despite being anucleate, platelets retain a diverse and functionally relevant repertoire of RNA species, some of which are capable of being translated independently of the parental megakaryocyte [12]. Platelet RNA sequencing studies have identified approximately 9500 protein‐coding genes, followed by noncoding small RNAs, rRNAs, pseudogenes, intergenic RNAs, abundant unannotated intronic and intergenic RNAs, and a multitude of repeats. This comprehensive profiling highlights the complexity and regulatory potential of the platelet transcriptome [13].

This insight into platelet RNA demonstrates the unique scale and composition of RNA within these cells compared with other blood components. Teruel‐Montoya et al. [14] reported that individual platelets carry an average of 2.2 femtograms of total RNA—approximately 1000 times less than the RNA content of a single leukocyte. However, given the considerably higher abundance of platelets in peripheral blood compared with leukocytes, their cumulative contribution to the overall blood RNA remains noteworthy (e.g., 0.88 million fg from 400 000 platelets versus 10 million fg from 5000 leukocytes per microliter of blood). Nevertheless, the final RNA yield and composition in liquid biopsy samples and subsequent differential expression analyses may be strongly influenced by the platelet isolation method employed [13]. Even minimal retention of any other blood cell types can significantly confound analyses of the platelet samples and will profoundly affect the analyzed liquid biopsy transcriptome profile and the interpretability of RNA‐based analyses [15].

A prospective multicenter STRIDE study by Banerjee et al. analyzed the impact of three platelet isolation protocols on next‐generation bulk RNA sequencing and functional assays. Leukocyte depletion—using anti‐CD45 beads or PALL™ filters as opposed to platelet washing alone—yielded a sufficient quantity of RNA for next‐generation sequencing and comparable sequencing quality metrics. As expected, the stringent removal of leukocytes led to lower relative expression of known leukocyte‐specific genes and higher relative expression of known platelet‐specific genes. Furthermore, while all three isolation techniques did not affect basal platelet reactivity, the anti‐CD45 bead isolation method reduced agonist‐induced integrin αIIbβ3 activation compared with washing alone [7, 16].

While platelets represent a valuable source of biological information, we hypothesized that the improved accuracy of several machine learning (ML) classifiers [2, 8, 17, 18] may in part be influenced by RNA signals originating from leukocytes retained in sample preparation, rather than being exclusively derived from platelets. Although the washed platelets protocol from the STRIDE study is not identical to thromboSeq applied in [8] study, both rely on similar centrifugation‐based steps for platelet enrichment and lack explicit leukocyte depletion, thus likely sharing a comparable risk of leukocyte‐derived RNA retention. According to the STRIDE study, washed platelets yielded higher RNA compared with bead‐isolated and filtered platelets, suggesting that leukocytes may contribute additional RNA content to these samples. This phenomenon was also confirmed by RNA‐seq analysis, where washed platelets demonstrated a higher number of RNA raw reads (*N* = 15 266) compared with filtered (*N* = 8722) and bead‐purified platelets (*N* = 11 126) [16].

In this study, we leveraged the largest, publicly available platelet RNA‐seq dataset generated to date, selecting 2351 samples [8] processed using the well‐established thromboSeq washed platelet protocol [7], to rigorously evaluate the extent to which leukocyte‐derived RNA contributes to the observed transcriptomic profiles. Given that this dataset serves as a cornerstone for many platelet RNA studies, it is critical to understand how inadvertently retained leukocytes may influence molecular classification and interpretation. To strengthen our analysis, we incorporated new, unpublished single‐cell and single‐platelet RNA‐sequencing data from EDTA blood derived from ovarian cancer patients, breast cancer patients, and control subjects, providing a valuable reference point for distinguishing true platelet‐derived transcripts from noise potentially arising from residual leukocyte‐associated RNA retention.

To this end, we addressed the following specific questions: (1) how can leukocyte‐associated RNA retention be estimated in platelet RNA‐seq *in silico*; (2) to what extent does leukocyte‐associated RNA retention introduce transcriptional noise into platelet RNA profiles; (3) is leukocyte‐associated RNA retention primarily driven by biological variability among patients or by technical factors during sample processing; (4) how does leukocyte‐associated RNA retention influence the performance of ML models trained on platelet transcriptomic data; and (5) can the effects of leukocyte‐associated RNA retention be mitigated retrospectively in already sequenced datasets.

By systematically dissecting the leukocyte contribution, our work not only reinforces the foundation for platelet‐based diagnostics but also evaluates the role of leukocyte transcriptomics in liquid biopsy‐based cancer classification—an essential and previously underexplored dimension.

## 2 Materials and methods

### 2.1 Processing of pan‐cancer data

Publicly available platelet RNA‐sequencing data were obtained from GSE183635, reported by In't Veld et al. [8]. Blood samples were collected between 2015 and 2021 at 13 participating medical institutions in Europe and the United States. The experiments were undertaken with written informed consent of each participant. The study was approved by the institutional review board and ethics committee at each participating hospital and conducted in accordance with the Declaration of Helsinki [8]. Following the methodology outlined by Pastuszak et al. [2], we normalized gene expression data using the DESeq2 package (version 1.44.0) [25] in R (version 4.4.1) through variance‐stabilizing transformation (VST) [26]. The human reference genome hg19 was used for annotation throughout. All samples underwent standardized preprocessing and were normalized collectively. For transcript selection, we applied the criteria described in [8], retaining 5346 transcripts with at least 30 reads in a minimum of 10% of samples in at least one group. The final dataset comprised 2351 platelet RNA samples.

### 2.2 Single‐cell RNA sequencing of PBMCs

Biospecimen collection was approved by the Independent Bioethical Committee for Scientific Research at Gdańsk Medical University (NKBBN 94/2017, NKBBN 748/2019‐2020). Biological samples are obtained exclusively from patients who have provided informed consent, in accordance with the principles of the Declaration of Helsinki (1983). Blood samples for the single‐cell cohort were collected between 2022 and 2024 at the Medical University of Gdańsk.

Whole blood was collected into 10 mL EDTA tubes before any treatment. It was then transferred to 15 mL Falcon tubes precoated with DB buffer. An equal volume of PBS was added, and the mixture was layered onto Histopaque and centrifuged (400 ***g***, 30 min, room temperature). The resulting buffy coat was placed on ice, centrifuged again (300 ***g***, 5 min, 4 °C), and the supernatant was discarded. The pellet was resuspended in a dedicated medium. After adding 0.5 mL of freezing medium, the cells were counted using a Countess cell counter (Thermo Fisher Scientific, Waltham, MA, USA), transferred to cryotubes, and stored at −80 °C until single‐cell sequencing was performed. Thawed buffy‐coat material was used for single‐cell sequencing, with a target of 10 000 cells per patient. No platelet‐enrichment step was performed.

Ovarian cancer and benign gynecologic control samples were processed at the Institute of Bioorganic Chemistry, Polish Academy of Sciences, in Poznań. Cryovials were thawed in a 37 °C water bath, and cells were transferred to 50‐mL conical tubes using wide‐bore pipette tips. After dilution in a warm 10% FBS complete growth medium, cells were centrifuged and resuspended. Cell concentration and viability were assessed with trypan blue staining using the Countess™ 3 FL Automated Cell Counter. Samples with viability over 70% (average 84%) were resuspended in 1X PBS + 0.04% BSA to a final concentration of 700–1200 cells/μl. Single‐cell libraries were prepared using the Chromium Next GEM Single Cell 3′ Reagent Kit (10× Genomics, Amsterdam, the Netherlands) following the manufacturer's protocol. Breast cancer and healthy‐donor samples were processed by Singleron Biotechnologies (B.V. Cologne, Germany). The protocol mirrored that of the Poznań site, with similar cell viability obtained, but the library preparation was performed using the SCOPE‐chip® micro‐well microfluidics system according to the manufacturer's instructions. Regardless of the processing site, all samples were sequenced on the NovaSeqX platform, in separate runs, targeting 20 000 reads per cell.

The single‐cell libraries were generated from buffy‐coat/PBMC material after density‐gradient processing. Platelet‐classified droplets in this dataset were co‐isolated during this workflow and were not generated through a dedicated platelet‐enrichment protocol. We therefore use the single‐cell data to define a leukocyte‐associated RNA signal detectable in platelet RNA‐seq libraries, while recognizing that these droplets do not represent a comprehensive platelet atlas.

### 2.3 Processing of single‐cell RNA‐seq data

Sequencing data were demultiplexed and converted to FASTQ format using CellRanger mkfastq. Unique molecular identifier (UMI) count matrices representing raw gene expression data were generated using the standard cell ranger software (version 5.01) from 10× Genomics, with the GRCh38 human reference genome employed for mapping. This processing involved filtering out low‐quality cells and removing apoptotic cells. Specifically, cells with fewer than 1000 reads overall, cells and platelets expressing fewer than 100 unique genes, and cells with over 10% mitochondrial RNA were excluded from further analysis. Since platelets are anucleate and contain low amounts of RNA, this unique‐gene threshold is stringent for platelet‐classified droplets; therefore, the number and proportion of platelet‐classified droplets retained after QC are reported in the Supporting Information in Table S1. Following quality control, each read count matrix was normalized to the total expression, scaled by a factor of 10 000, and log‐transformed. Single‐cell and platelet sequencing analysis was performed in r (version 4.4.1), with the seurat library (version 5.1.0) [27]. Canonical platelet and leukocyte transcripts were defined according to the markers listed in PanglaoDB [28].

We added UMAP visualizations colored by cell type and sample (Fig. S1,B) with markers used for detection (Table S2), per‐sample platelet‐classified droplet QC summaries (Table S1), and pseudobulk platelet comparisons (Fig. S2A,B) to the Supporting Information to increase annotation transparency. We did not use integration methods, such as Harmony or Seurat integration as the primary basis for marker discovery, because integration can obscure platform‐specific detection and dropout patterns that are directly relevant to evaluating whether transcripts are detected in platelet‐classified droplets.

### 2.4 Estimation of leukocyte‐associated RNA retention level

For cell‐type classification using ucell library in single‐cell dataset (version 2.8.0) [29], we employed a panel of cell‐type‐specific marker genes: PDGFRB, FAP (fibroblasts), PTPRC, CD3E, CD3D, CD3G, CD4, CD68, CD8A, CD8B, CD14, CD19, KLRF1, NCAM1, IL7R, SELL, LYZ, S100A9, CD69, MS4A1, CD74, CD96, CD2, CD52, CD53, CD55, FCN1, FCGR3B, FCGR3A, CCR7, CD27, IL2RA, FOXP3, TIGIT, FUT4, CEACAM8, ITGAM, ITGB2 (leukocytes), PPBP, ITGB3, PF4, GP9, ITGA2B, CD63, SELP, TUBB1, CCL5 (platelets), and FCER1A, CD1C, AXL, and CD83 (dendritic cells) [28]. Following cell‐type classification and expression normalization, we identified, for each sample, genes with zero cumulative expression in platelet‐classified droplets and a cumulative expression in leukocytes equal to or exceeding 75% of the total number of cells classified as leukocytes for that sample. Genes meeting both criteria in at least nine samples were retained as candidates. To evaluate robustness of the discovery procedure, the analysis was repeated with a relaxed platelet‐expression tolerance (< 1% cumulative expression in platelet‐classified droplets) while preserving the leukocyte‐recurrence requirement. Both the strict (zero platelet expression) and relaxed criteria identified the identical set of 138 candidate leukocyte‐associated genes. Because the bulk platelet RNA‐seq dataset had been processed in the hg19/GRCh37 annotation while the single‐cell data used GRCh38, strict gene‐level harmonization were applied. Six genes (NOP53, ATP5MC2, RSRP1, TRBC1, ATP5MG, and SARAF) could not be mapped reliably and were excluded, leaving a final panel of 132 genes. For each bulk platelet RNA‐seq sample, raw counts of these 132 genes were converted to counts per million (CPM) by multiplying by 1000,000 and dividing by the total library size. The leukocyte‐associated RNA retention score was calculated as the sum of the CPM values across the 132 genes. Because CPM normalization accounts for library size but not transcript length, the score is interpreted as a compositional measure of RNA abundance.

In addition to the primary 132‐gene score, a PTPRC‐only score was computed. A nonribosomal sensitivity score was also generated by first removing 23 ribosomal‐protein genes from the 138‐candidate panel, yielding 115 nonribosomal genes. After exclusion of the six nonmappable genes, the final nonribosomal score was based on the remaining 109 genes. Comprehensive definitions of each gene subset are provided in Table S3.

### 2.5 Analysis of leukocyte‐associated RNA retention determinants

To investigate factors associated with leukocyte‐associated RNA retention, we used a random forest regression (RFR) model to predict the final 132‐gene retention score in the platelet pan‐cancer dataset. The model included sex, age, cancer stage or healthy‐donor status, blood collection center, library size, and cancer type. The random forest model was used due to the nonlinearity of the leukocyte score. We used the default hyperparameters.

### 2.6 Threshold‐based clustering of leukocyte‐associated RNA retention

Our next objective was to identify subsets of samples in the platelet pan‐cancer dataset characterized by low, medium, or high‐leukocyte‐associated RNA retention. This grouping was intended as a descriptive and analytical stratification of an RNA‐composition axis, not as a claim of physical platelet purity. Samples were categorized into three groups based on the estimated retention score, and thresholds were selected using a grid‐search procedure that maximized the Caliński–Harabasz index. The metric was selected to ensure the formation of clusters that are both well‐separated and relatively balanced in size.

### 2.7 Sample matching

To evaluate the effect of leukocyte‐associated RNA retention on ML model performance under controlled conditions, we constructed a balanced dataset containing equal numbers of low‐ and high‐leukocyte‐retention samples. Starting from the full cohort of 2351 platelet RNA‐seq samples, we excluded samples with intermediate leukocyte‐associated RNA retention scores, samples with missing information on sex or age, and samples from individuals with nonmalignant conditions. Only healthy donors and patients diagnosed with cancer were retained for the binary cancer‐detection task. Because the ‘patient condition’ variable contained 19 categories (healthy donors plus 18 distinct cancer types), we performed matching separately within each condition stratum using age (seven categories), sex (two categories), cancer stage (six categories), and blood collection center (13 centers) as matching variables. Matched subsets from all strata were subsequently merged. This yielded a final dataset of 754 samples comprising 377 low‐retention and 377 high‐retention samples. Three cancer types (breast cancer, renal cell carcinoma, and hepatocellular carcinoma) could not be included, as they contained only low‐retention samples; the matched dataset therefore encompassed 15 cancer types in addition to healthy donors. By design, cancer type (including healthy status) was perfectly balanced between the low‐ and high‐retention groups. Substantial balance was also achieved for age, sex, and cancer stage. Blood collection center could not be fully balanced because center identity was partially confounded with leukocyte‐associated RNA retention levels. Residual center imbalance is therefore reported transparently (Table S4).

### 2.8 Training, validation, and testing split

To ensure balanced representation of low‐ and high‐retention samples across training, validation, and testing sets, we implemented a stratified data‐splitting strategy based on the matched dataset. The dataset was initially partitioned into a testing set (60%) and a training‐validation set (40%), with stratification by leukocyte‐associated RNA retention group (high‐ or low‐retention), patient condition (healthy or one of 15 cancer types), and cancer stage. For samples with unique combinations of these variables, stratification was performed solely by the leukocyte‐associated RNA retention group to maintain the 60/40 split. Subsequently, the training‐validation set was divided equally into training and validation sets (a 50/50 ratio), using the same stratification criteria. Samples with unique variable combinations were again stratified by leukocyte group only. To enhance the robustness of model evaluation, this stratified splitting procedure was repeated 10 times using different random seeds (from 0 to 9), and the results were averaged. As a result, the average number of high‐ and low‐leukocyte samples in the training and validation sets was approximately 75 each, while the test set contained on average 225 low‐leukocyte samples and 227 high‐leukocyte samples.

### 2.9 Machine learning models

To assess the impact of leukocyte‐associated RNA retention on machine‐learning‐based cancer detection, we employed two complementary strategies. We trained and evaluated models on the matched low‐ versus high‐retention dataset under strongly contrasted retention conditions. We implemented 10 diverse classifiers for the binary malignancy detection task: logistic regression, support vector machine (SVM), k‐nearest neighbors (KNN), random forest, balanced random forest, extra trees, XGBoost, LightGBM, gradient boosting, and AdaBoost. Hyperparameters for each classifier were optimized independently using the optuna python library (version 4.0.0) [30] on the matching dataset. A dedicated Optuna study was performed for every model, in which hyperparameters were sampled from predefined search spaces, the classifier was trained on the training set, and performance was evaluated on the validation set using ROC AUC (calculated from predicted probabilities) as the optimization objective. Test‐set ROC AUC was recorded for reporting but was withheld from the hyperparameter optimization process. The hyperparameter families are detailed in the Supporting Information in Table S5. The complete set of hyperparameter ranges is available in our GitHub repository (see the Data availability statement section). We also evaluated whether addition of the final 132‐gene leukocyte‐associated RNA retention score materially altered cancer‐detection performance of a logistic‐regression classifier when the full cohort of 2351 samples is used.

### 2.10 Leukocyte‐associated RNA retention correction

To evaluate the influence of leukocyte‐associated RNA retention as a sensitivity analysis (rather than as a universal correction step), we applied two complementary strategies for modeling and mitigating retention‐related variation. First, we applied the remove unwanted variation (RUV) method [31] to estimate and adjust for unwanted variation. The key part of the RUV algorithm is the identification of negative control genes that are unaffected by the primary variable of interest (cancer status) but influenced by the confounding factor (leukocyte score). These genes were selected based on two criteria: (1) an adjusted *P*‐value > 0.5 from a likelihood ratio test for malignancy, indicating no association with cancer status, and (2) a Spearman correlation coefficient > 0.6 with leukocyte score, reflecting sensitivity to leukocyte‐associated RNA retention. The RUV algorithm used these genes to estimate unwanted variation and adjust the data accordingly, reducing its impact on downstream analyses.

To complement the RUV method, we developed a second deconvolution approach to correct for leukocyte‐associated RNA retention in platelet RNA‐sequencing data by estimating and subtracting leukocyte‐derived reads. Assuming that total reads comprise contributions from platelets, leukocytes, and noise, we posit that leukocyte‐specific transcript abundance is proportional to leukocyte enrichment, approximated as follows: Reads from leukocytes ≈ Gene‐specific coefficient × Expression of leukocyte markers. To estimate this coefficient, we select 20% of healthy‐donor samples with the lowest and highest leukocyte scores and pair each low‐leukocyte‐score sample with each high‐leukocyte‐score sample to minimize individual biological differences. For each pair, we calculate the difference in gene expression (*d*) between the high‐ and low‐leukocyte‐score samples for a given gene, using the leukocyte score of the high‐leukocyte‐score sample as the denominator (*c*), yielding a gene‐specific coefficient (*x* = *d*/*c*). By averaging *x* across all pairs, we further reduce individual biological variability. This coefficient is then multiplied by the leukocyte score of each sample and subtracted from its observed gene expression to obtain corrected expression levels across all samples. This correction is applied to genes with a *P*‐value < 0.05 from a Wilcoxon test comparing high‐ versus low‐leukocyte‐score samples, ensuring effective adjustment of leukocyte‐influenced genes while preserving relevant biological signals and minimizing overcorrection.

### 2.11 Computational packages

All analyses were conducted using r (version 4.4.1) and python (version 3.12.2). Heatmaps were generated in r using the pheatmap package (version 1.0.12), while scatter plots were produced in Python with the matplotlib (version 3.8.3) and seaborn (version 0.13.2) libraries. Data storage and manipulation were handled using pandas (version 2.2.1). Sample matching was performed using the MatchIt package in r (version 4.7.1). Effect size calculations for the Mann–Whitney *U*‐test were conducted using the rstatix package (version 0.7.2). ML workflows, including classification and regression modeling, metric computation, principal component analysis (PCA), and data partitioning (training, validation, and testing), were implemented using Python's scikit‐learn (version 1.5.2) and imbalanced‐learn (version 0.12.3) libraries. For removal of unwanted variation (RUV), we utilized the built‐in implementation from the RUVSeq package (version 1.38.0) in r. All statistical tests were carried out using either Python's scipy library (version 1.11.4) or r's stats package (version 4.4.1). Statistical significance was defined as *P* < 0.05.

## 3 Results

### 3.1 Surveying leukocyte‐associated RNA retention in platelet RNA samples

In this study, we analyzed publicly available bulk platelet pan‐cancer data (GEO: GSE183635), comprising 2351 samples from asymptomatic and symptomatic controls, as well as 18 cancer types. In addition, we utilized a single‐cell liquid biopsy dataset containing both platelet‐classified droplets and leukocytes, which included samples from 10 breast cancer patients (2 luminal B, HER2‐negative; 2 luminal B, HER2‐positive; and 6 triple‐negative), 12 high‐grade ovarian cancer patients, 4 patients with nonmalignant conditions (genital cord tumor, Brenner tumor, endometriosis, and Borderline tumor), and 3 healthy donors. The single‐cell analysis identified a 138‐gene candidate leukocyte‐associated marker panel. After strict cross‐resource harmonization between the single‐cell and bulk annotation contexts, 132 genes were retained for the final bulk leukocyte‐associated RNA retention score. Using this gene panel, we estimated leukocyte‐associated RNA retention in the bulk platelet pan‐cancer dataset.

Subsequently, based on the Calinski–Harabasz index, we classified samples into high‐ and low‐leukocyte groups. To minimize confounding factors, we created a truncated matching dataset in which age, sex, cancer stage, blood collection unit, and cancer type were relatively balanced between the high‐ and low‐leukocyte groups. This matched dataset was then split into training, validation, and testing sets, ensuring balance of leukocyte groups, cancer stages, and cancer types across all sets.

Finally, we evaluated the impact of leukocyte‐associated RNA retention on cancer classification using ML models. Both training and testing sets were analyzed. To increase robustness, we employed 10 different random splits for data partitioning and 3 different random seeds for each model. Figure 1 presents a general overview of the study.

**Fig. 1:**
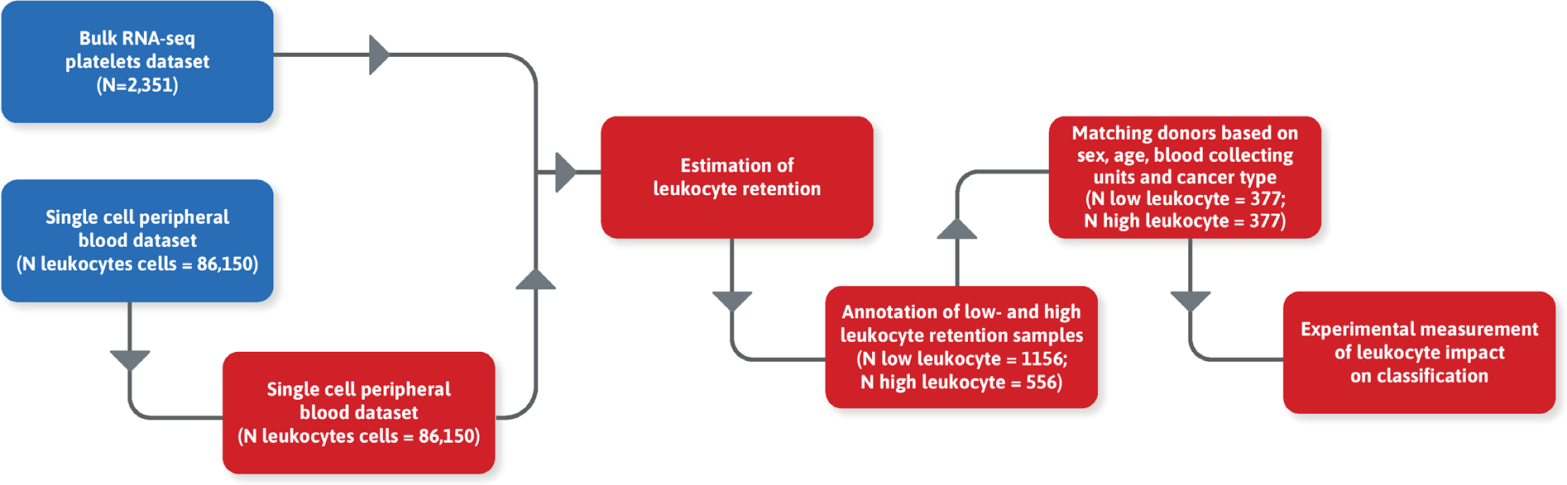
General overview of the study. Blue blocks represent datasets and red blocks represent data manipulations.

### 3.2 Estimation of leukocyte‐associated RNA retention level

The leukocyte‐associated RNA retention level was estimated using a curated 138‐gene set detailed in Table S6. This gene set included RNA transcripts highly enriched in leukocytes compared with platelets, specifically leukocyte‐associated genes (e.g., PTPRC, CD3E, CD3G, CD3D, CCR7, IL7R, and CD2), as well as several ribosomal genes (e.g., RPL12, RPL24, RPL27, RPL36, RPL37A, RPL6, RPS11, and RPS13). Figure S3 presents a heatmap of the expression of these genes in the single‐cell dataset, aggregated into pseudobulk profiles by summing the raw counts from individual cells of the same type—either leukocytes or platelets—into a single composite sample. The heatmap reveals significantly lower expression of these genes in platelets. The patient's paired Wilcoxon *P*‐value for platelets and leukocyte CPM‐normalized expression sum was approximately 7.63e‐6, indicating statistically significant differences between platelets and leukocytes. The relaxed < 1% platelet‐expression sensitivity analysis recovered the same 138 candidate genes as the original zero‐expression criterion. After strict gene‐level harmonization between the GRCh38 single‐cell annotation and the hg19/GRCh37 bulk platelet RNA‐seq annotation, 132 genes were retained for the final operational bulk score. The final retention score was strongly aligned with both the PTPRC‐only score (Fig. S4; Pearson's *r* = 0.831, *P* < 0.001; Spearman's ρ = 0.913, *P* < 0.001) and the mapped 109‐gene nonribosomal sensitivity score (Fig. S5; Pearson's *r* = 0.967, *P* < 0.001; Spearman's ρ = 0.990, *P* < 0.001), indicating that the retention axis is robust across alternative marker definitions and is not substantially affected by ribosomal‐gene concerns.

### 3.3 Impact of leukocyte‐associated RNA retention on platelets pan‐cancer data

Leukocyte‐associated RNA retention was quantified by summing the CPM‐normalized expression of the selected 132 genes for each sample in the platelets pan‐cancer dataset. To evaluate its impact on data structure, we applied PCA to transform the data into a two‐dimensional projection, examining clustering patterns based on leukocyte‐associated RNA retention scores, patient condition (malignant or nonmalignant), cancer stage (I, II, III, IV, stage unknown or healthy), and blood collection institute. Figure 2–D illustrate these projections. A clear gradient is observed for leukocyte‐associated RNA retention, with samples exhibiting the lowest leukocyte signal positioned on the left and those with the highest signal on the right, indicating a strong influence of leukocyte‐associated RNA retention on data clustering. In contrast, no distinct patterns were observed for cancer type, cancer stage, or blood collection institute, suggesting that leukocyte‐associated RNA retention is a more dominant factor in differentiating samples than these variables. PCA revealed that leukocyte‐derived signals dominate the first principal component, accounting for the greatest variance across samples and overshadowing biological variables, such as age, sex, or cancer stage. This variance appeared largely independent of these biological characteristics and diminished in higher‐order components (Fig. S6A,B), suggesting that platelet‐specific signals remain robust and distinguishable.

**Fig. 2:**
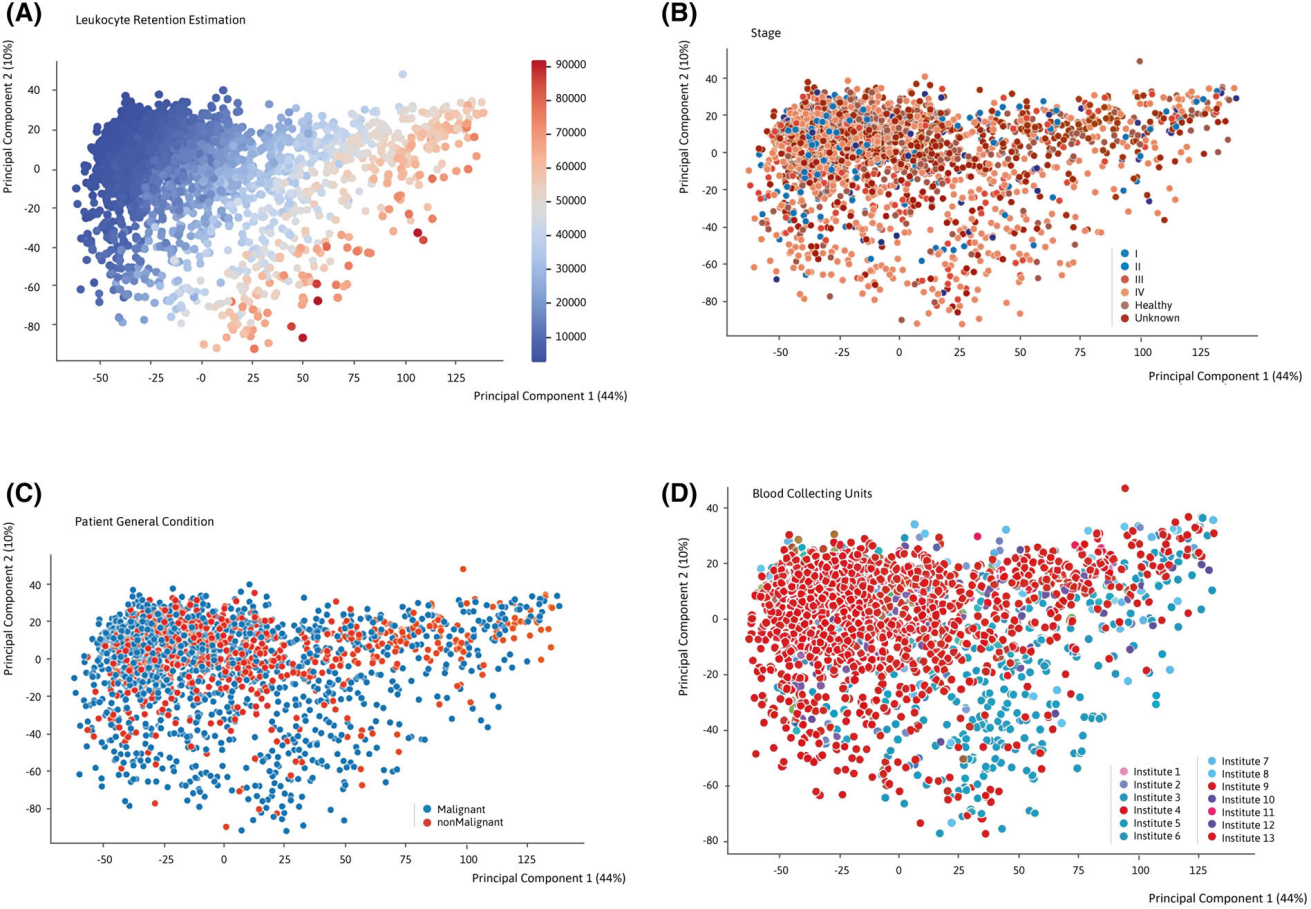
(A–D) Principal component analysis (PCA) projections of the platelets pan‐cancer dataset (*N* = 2351), colored by (A) leukocyte‐associated RNA retention scores, (B) patient condition (malignant or nonmalignant), (C) cancer stage (I, II, III, IV, healthy, or unknown), and (D) blood collection institute.

### 3.4 Analysis of leukocyte‐associated RNA retention determinants

To identify factors contributing to leukocyte‐associated RNA retention, we developed a RFR model to predict leukocyte‐associated RNA retention scores, achieving an *R* ^2^ of 0.90, which indicates that the model explains 90% of the variance in the data. Feature importance analysis revealed the following contributions: sequencing library size (0.38), blood collection institute (0.33), age (0.20), cancer stage (0.06), sex (0.03), and information if a patient has cancer or not (0.01). To further analyze these effects, we trained two additional RFR models: one using biological factors (age, sex, cancer stage, and information if a patient has cancer or not) and another using technical factors (blood collection institute and library size). The biological model yielded an *R* ^2^ of 0.22, while the technical model achieved an *R* ^2^ of 0.87, indicating that technical factors have a greater influence on leukocyte‐associated RNA retention than biological factors. We further assessed the association between the leukocyte‐associated RNA retention score and library size (Fig. S7), observing a moderate inverse correlation (Pearson's *r* = −0.362, *P* < 0.001; Spearman's ρ = −0.479, *P* < 0.001). Because this trend is opposite to what would be expected if higher sequencing depth were simply inflating the retention score, library size alone is unlikely to explain the observed retention signal. These analyses support the interpretation that retention is strongly associated with pre‐analytical and technical structure, although biological and inflammatory contributions cannot be excluded.

### 3.5 Threshold‐based clustering of leukocyte‐associated RNA retention

To categorize samples by leukocyte‐associated RNA retention levels, we determined optimal thresholds using cluster analysis. The threshold separating low and medium retention samples was 11 600, and the threshold separating medium and high‐retention samples was 33 100, with leukocyte scores ranging from 2529.34 to 90667.13. This clustering achieved a Calinski–Harabasz score of 661.90, indicating robust separation. Figure 3 presents a PCA plot visualizing the resulting clusters: low retention (1156 samples), medium retention (639 samples), and high retention (556 samples). Approximately 24% of the dataset exhibited high‐leukocyte‐associated RNA retention.

**Fig. 3:**
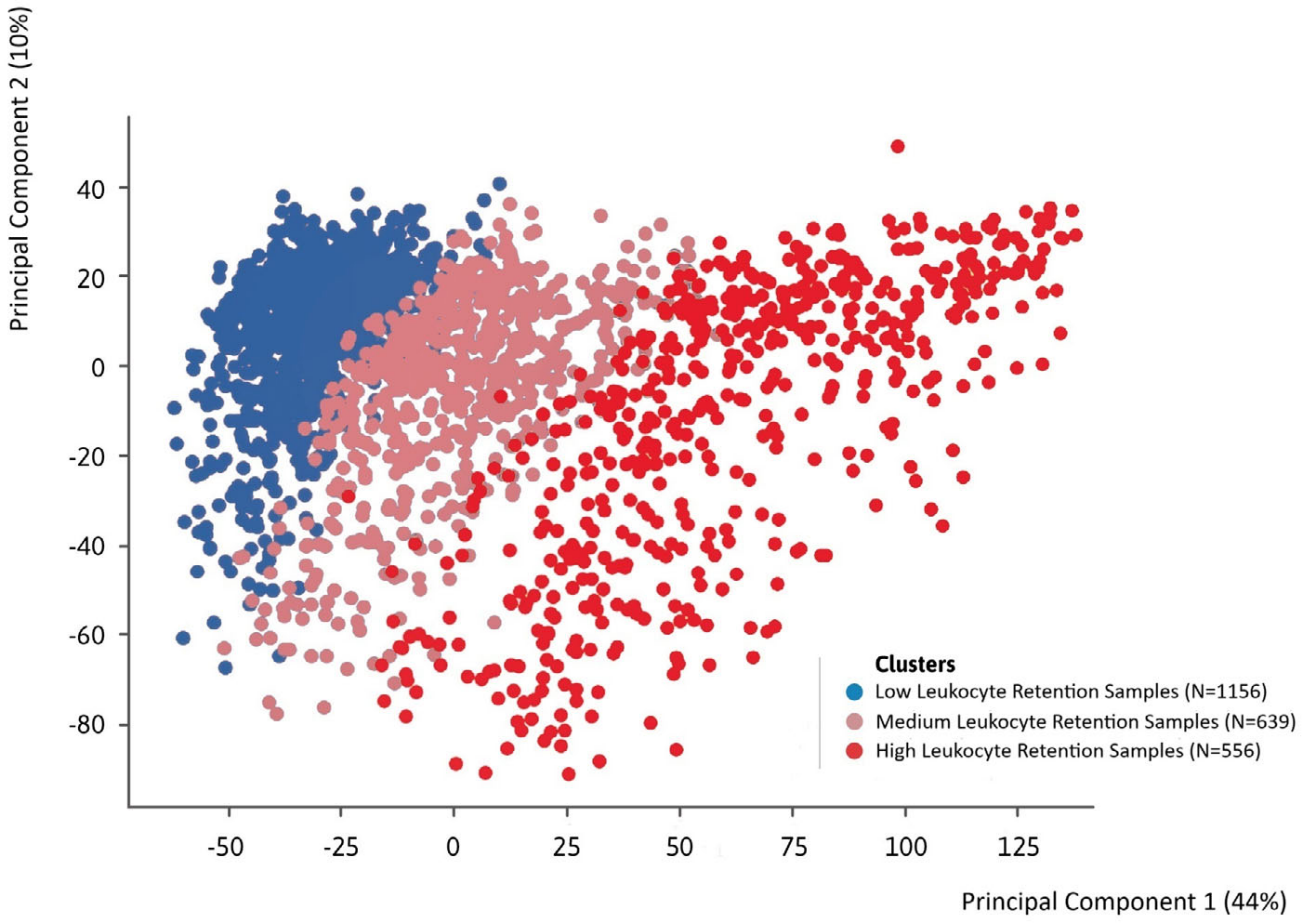
Principal component analysis (PCA) projection of platelets pan‐cancer dataset with optimal division into three clusters based on leukocyte‐associated RNA retention level (low‐leukocyte‐associated RNA retention samples—*N* = 1156; medium leukocyte‐associated RNA retention samples—*N* = 639; high‐leukocyte‐associated RNA retention samples—*N* = 556).

### 3.6 Sample matching

Next, we performed sample matching to create a truncated dataset with the same number of low and high‐leukocyte samples and with balanced factors, such as age, sex, cancer stage, cancer type, and blood collecting institute. The purpose of such matching is to prepare a reliable framework for ML experiments and to reduce the influence of aforementioned factors. As a result, we obtained a dataset with 754 samples: 377 low and high‐leukocyte records. Table S4 presents the concrete distribution of age, sex, cancer stage, cancer type, and blood collecting units in this matching dataset.

### 3.7 Primary matched low/high analysis and machine learning experiments

To assess the effect of leukocyte‐associated RNA retention on the platelet RNA‐based classification, we evaluated a limited but diverse set of ML algorithms, commonly used in classification tasks. As a first step, we aimed to assess how models trained on both low‐ and high‐retention samples perform on the corresponding, separate low‐ and high‐retention test set in order to evaluate the impact of retention on model performance during testing. The model performed a binary classification task of detecting cancer in a liquid biopsy sample (classification ‘healthy’ versus ‘cancer’). Table 1 summarizes the evaluation results of the ML models. Each model was trained and evaluated three times using different random seeds (7, 42, and 123). For each of these runs, 10 distinct training‐validation‐test splits (with independent seeds from 0 to 9) were employed. As a result, each reported performance metric represents the average over 30 model runs and is expressed as the mean ± standard deviation.

**Table 1:** Performance of 10 classifiers across low‐ and high‐leukocyte‐retention test subsets. Area under the receiver operating characteristic curve (AUC ROC) for 10 different classifiers evaluated on the low‐ and high‐retention subsets of the test set and trained on the whole training set containing both low‐ and high‐retention samples. Each reported value represents the average performance over 30 independent model runs. Results are presented as the mean ± standard deviation. Table cells highlighted in green and red indicate values that are, respectively, higher and lower within the specified group. Additionally, cells containing FDR‐adjusted *P*‐values of the paired Wilcoxon test of less than 0.05 are highlighted in blue.

Model name	AUC ROC for the low‐retention test set (mean ± SD)	AUC ROC for high‐retention test set (mean ± SD)	FDR‐adjusted *P*‐value of paired Wilcoxon test
Logistic regression	0.89 ± 0.02	0.91 ± 0.01	0.0002
Support vector machine	0.91 ± 0.01	0.90 ± 0.02	0.0998
LightGBM	0.86 ± 0.02	0.87 ± 0.03	0.0998
AdaBoost	0.85 ± 0.02	0.86 ± 0.03	0.7283
Random forest	0.86 ± 0.02	0.85 ± 0.02	0.0998
Extra trees	0.85 ± 0.03	0.84 ± 0.03	0.0998
XGBoost	0.84 ± 0.02	0.83 ± 0.03	0.7457
Balanced random forest	0.84 ± 0.03	0.82 ± 0.03	0.0062
Boosting	0.84 ± 0.03	0.82 ± 0.03	0.1427
K‐nearest neighbors	0.82 ± 0.06	0.78 ± 0.03	0.0067

Among the evaluated models, only logistic regression, LightGBM, and AdaBoost achieved higher AUC scores on the high‐retention test set compared with the other models. However, in most cases, the differences in average performance between the high‐ and low‐retention sets were not statistically significant. Statistically significant differences were observed only for logistic regression, balanced random forest, and K‐nearest neighbors. These findings suggest that the impact of retention level on model performance varies across different models. Nevertheless, the differences in performance are generally minor and, in most cases, not statistically significant.

Next, we aimed to investigate the impact of retention during training. To this end, models were trained separately on the low‐ and high‐retention training set and evaluated on a test set containing both low‐ and high‐retention samples combined. Table 2 presents the results, which were also averaged over 30 independent runs and reported as mean ± standard deviation.

**Table 2:** Performance of 10 classifiers trained separately on low‐ and high‐leukocyte‐retention samples and evaluated on the complete test set. Area under the receiver operating characteristic curve (AUC ROC) for 10 different classifiers evaluated on the whole testing set containing both low‐ and high‐retention samples and trained on low‐ and high‐retention training sets separately. Each reported value represents the average performance over 30 independent model runs. Results are presented as the mean ± standard deviation. Table cells highlighted in green and red indicate values that are, respectively, higher and lower within the specified group. Additionally, cells containing adjusted *P*‐values of paired Wilcoxon test less than 0.05 are highlighted in blue.

Model name	AUC ROC for whole test set while training on low‐retention train set (mean ± SD)	AUC ROC for whole test set while training on high‐retention train set (mean ± SD)	FDR‐adjusted *P*‐value of paired Wilcoxon test
Logistic regression	0.82 ± 0.04	0.83 ± 0.03	0.5307
SVM	0.76 ± 0.06	0.84 ± 0.04	0.0003
LightGBM	0.74 ± 0.04	0.79 ± 0.03	0.0006
AdaBoost	0.77 ± 0.03	0.78 ± 0.04	0.5307
Random forest	0.76 ± 0.03	0.76 ± 0.04	0.5879
Extra trees	0.75 ± 0.04	0.76 ± 0.04	0.6408
XGBoost	0.71 ± 0.04	0.74 ± 0.02	0.0085
Balanced random forest	0.72 ± 0.04	0.73 ± 0.04	0.5879
Boosting	0.74 ± 0.03	0.75 ± 0.04	0.5307
KNN	0.71 ± 0.05	0.72 ± 0.03	0.5879

With respect to the influence of retention on model training, all models exhibited equal or improved performance when trained on the high‐retention set. However, statistically significant improvements were observed only for SVM, LightGBM, and XGBoost. These results indicate that training on high‐retention data may lead to modest performance gains; nonetheless, for most models, the differences compared with training on the low‐retention set are not statistically significant.

### 3.8 Leukocyte‐associated RNA retention correction

To assess transcriptome‐wide alterations while accounting for leukocyte‐associated RNA retention, we applied two correction methods. The first approach utilized the RUV algorithm. The concrete list of genes used for RUV as a negative control is presented in Table S7. The PCA plot, illustrating the data structure after RUV correction and DESeq normalization, is presented in Fig. 4. To evaluate the impact on sample clustering, three categories of leukocyte‐associated RNA retention—low, medium, and high—were annotated on the plots. Application of the RUV method resulted in a substantial alteration of the clustering structure, effectively eliminating the grouping of samples based on leukocyte‐associated RNA retention. This effect was quantitatively assessed using the Caliński–Harabasz index, which decreased markedly from 661.90 before correction to 12.20 postcorrection. This sharp decline indicates a significant loss of cluster separability associated with leukocyte‐associated RNA retention following the RUV adjustment. In addition, we also tested RUV based on library size, as in In't Veld et al. [8] in order to compare it to the proposed methods. The library size RUV‐correction threshold was the same as for the pan‐cancer‐detection algorithm. The method was also able to reduce leukocyte‐associated RNA retention drastically, scoring a Caliński–Harabasz index of 5.78. Figure S8 presents the 3D PCA plots before and after the correction. We also verify the effect of leukocyte‐associated RNA retention to SVM probabilities from In't Veld et al. [8], which was shown on Figs S9 and S10.

**Fig. 4:**
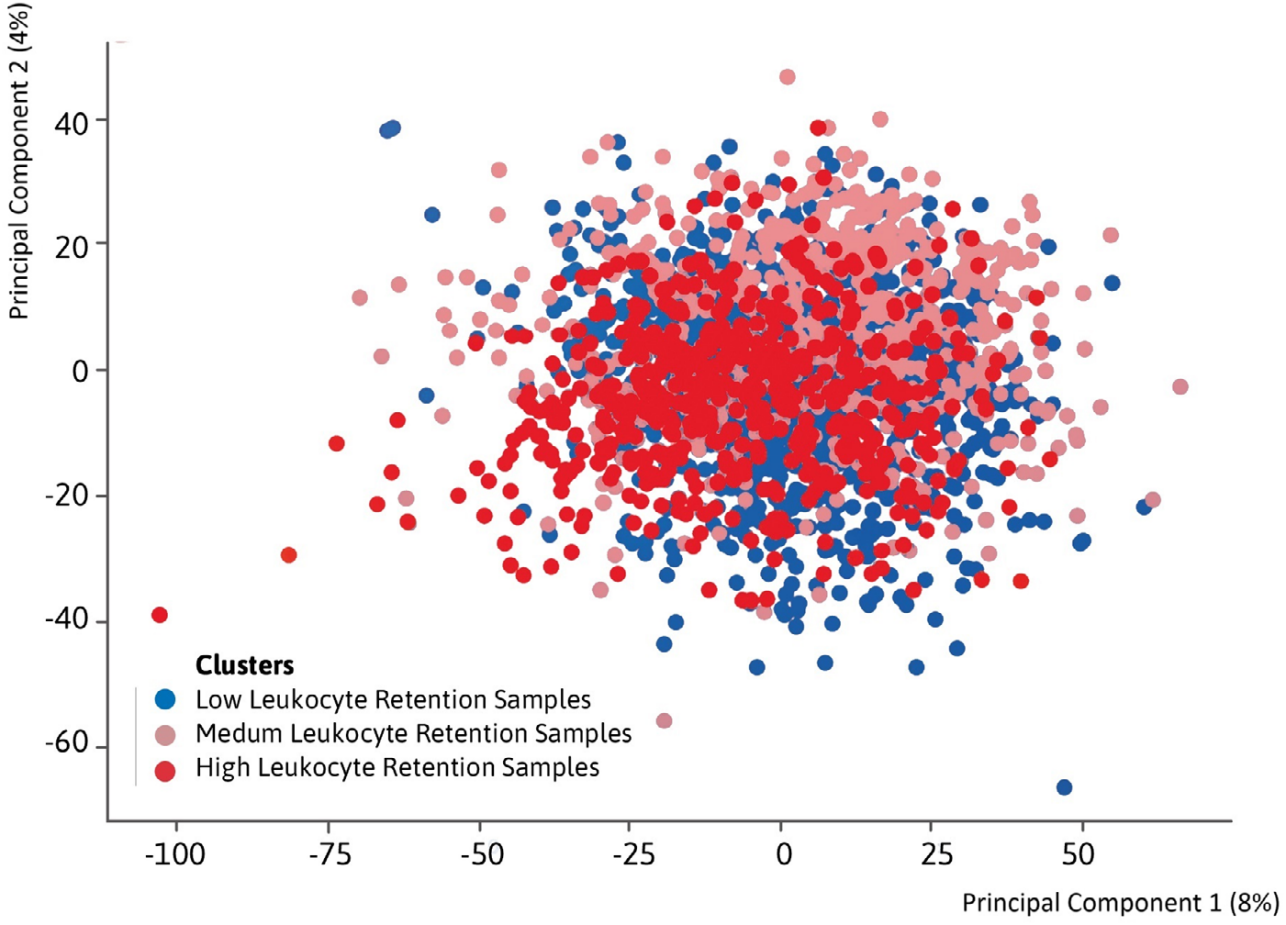
Principal component analysis (PCA) projections of the entire platelets pan‐cancer dataset, grouped by leukocyte‐associated RNA retention levels (low, medium, and high) after remove unwanted variation (RUV) correction based on leukocyte score (low‐leukocyte‐associated RNA retention samples—*N* = 1156; medium leukocyte‐associated RNA retention samples—*N* = 639; high‐leukocyte‐associated RNA retention samples—*N* = 556).

Next, we applied the linear leukocyte‐associated RNA retention correction model and Deseq normalization to the platelet dataset to reduce the impact of leukocyte‐associated RNA retention on the data. As shown by the PCA in Fig. 5, this approach attenuated—but did not eliminate—the influence of leukocyte‐derived signals. The Calinski–Harabasz index decreased initially from 661.90 before linear correction to 99.49 after the correction, indicating that the data's ability to cluster reduced significantly.

**Fig. 5:**
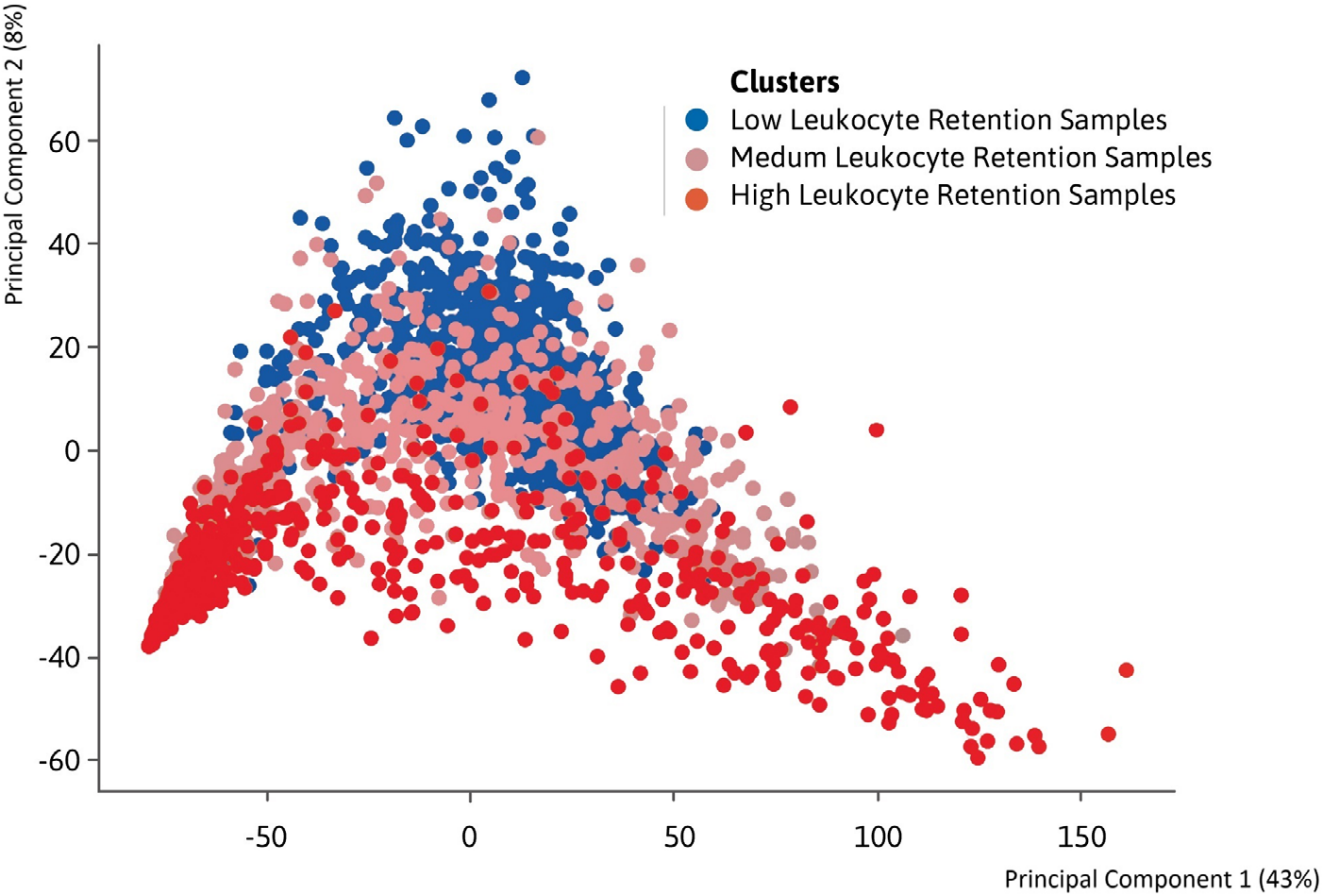
Principal component analysis (PCA) projections of the entire platelets pan‐cancer dataset, grouped by leukocyte‐associated RNA retention levels after linear correction (low‐leukocyte‐associated RNA retention samples—*N* = 1156; medium leukocyte‐associated RNA retention samples—*N* = 639; high‐leukocyte‐associated RNA retention samples—*N* = 556).

Furthermore, we evaluated the performance of the correction algorithms on the matching dataset, training on both low‐ and high‐leukocyte‐associated RNA retention samples combined and testing separately for low‐ and high‐retention samples. The results were also averaged out over 30 runs. The results for the logistic regression model are presented in Table 3. Notably, the RUV‐corrected data still exhibit statistically significant differences between the two groups, especially for the method based on library size from In't Veld et al., [8]. In contrast, the linear correction yields comparable results across both test sets, with no statistically significant difference observed. Although the linear correction method shows a slight decrease in overall performance, the lack of substantial group‐wise differences suggests that it achieves more effective correction.

**Table 3:** Performance of leukocyte‐associated RNA retention correction methods. Area under the receiver operating characteristic curve (AUC ROC) for three leukocyte‐associated RNA retention correction methods evaluated on the low‐ and high‐retention subsets of the test set using the logistic‐regression model. The results were averaged out over 30 runs. Each model was trained on the whole training set containing both low‐ and high‐retention samples. Table cells highlighted in green and red indicate values that are, respectively, higher and lower within the specified group. Additionally, cells containing adjusted *P*‐values from the paired Wilcoxon test of less than 0.05 are highlighted in blue.

	AUC ROC for the low‐retention test set (mean ± standard deviation)	AUC ROC for high‐retention test set (mean ± standard deviation)	Adjusted *P*‐value of paired Wilcoxon test
Without correction	0.89 ± 0.02	0.91 ± 0.01	0.0002
Leukocyte‐retention‐based RUV correction (proposed)	0.90 ± 0.02	0.92 ± 0.01	0.001
Library‐size‐based RUV correction	0.85 ± 0.02	0.92 ± 0.01	5.588e‐9
Linear correction (proposed)	0.88 ± 0.02	0.88 ± 0.02	0.792

### 3.9 Full‐cohort classifier sensitivity analysis

In the full‐cohort classifier sensitivity analysis, which complemented the primary matched low/high analysis, the final 132‐gene leukocyte‐associated RNA retention score was included as a continuous covariate in the logistic‐regression cancer‐classification model across all 2351 samples. Adding the retention score changed the overall binary cancer‐detection AUC ROC only minimally, from 0.9247 to 0.9249. The retention‐score coefficient was small (−0.000065) and ranked 5336th of 5347 model features by absolute coefficient magnitude.

## 4 Discussion

Platelet‐derived RNA represents a promising avenue for liquid biopsy, offering a minimally invasive means of detecting, characterizing, and monitoring of cancers, including those at early stages [2, 7, 8, 18, 19]. Large‐scale studies of tumor‐educated platelets (TEPs) have demonstrated robust diagnostic performance, with high accuracy, sensitivity, and specificity across a variety of cancer types [7, 8]. However, leukocyte‐associated RNA retention during platelet isolation introduces transcriptional noise that may hinder biomarker discovery. Banerjee et al. [16] reported that platelet isolation via centrifugation steps, compared with more stringent methods, such as platelet filtering and CD45 depletion, might result in relatively elevated levels of leukocyte‐specific transcripts (e.g., PTPRC), identifying leukocyte‐associated RNA retention as a potential confounder of the platelet RNA‐based models. As most publicly available platelet RNA‐sequencing datasets—including cohort of 2351 samples—were generated using these standard protocols, assessing the impact of leukocyte‐associated RNA retention as essential. By leveraging single‐cell RNA‐sequencing data from ovarian and breast cancer patients, this study also investigated the measurement, extent, determinants, and consequences of leukocyte‐associated RNA retention, aiming to advance the accuracy of platelet‐based cancer diagnostics.

Marker discovery from the single‐cell blood‐fraction reference identified 138 candidate leukocyte‐associated genes characterized by zero or minimal expression in platelet‐classified droplets and high cumulative expression in leukocytes. After strict gene‐level harmonization between the GRCh38 single‐cell and hg19/GRCh37 bulk annotation contexts, 132 genes were retained for the final operational leukocyte‐associated RNA retention score. Leukocyte‐associated RNA retention in the bulk platelet samples was quantified by summing the CPM normalized expression values of these 132 genes. We observed heterogeneous, low‐level expression of some leukocyte markers in single platelets, which may reflect platelet sequestration of leukocyte‐derived RNAs, profiling of rare platelet–leukocyte aggregates, or limited expression by platelet subpopulations. A relaxed sensitivity analysis permitting < 1% cumulative expression in platelet‐classified droplets recovered the identical set of 138 candidate genes. The final retention score remained highly concordant with both a PTPRC‐only score and the mapped 109‐gene nonribosomal sensitivity score, supporting the robustness of the retention axis to marker definition and ribosomal‐gene concerns. Leukocyte‐associated RNA retention is quantitatively heterogeneous among the employed platelet RNA samples and significantly influenced platelet transcriptomic profiles, as PCA without any data correction method revealed that leukocyte‐derived signals dominate the first principal component, accounting for the most significant variance across samples and overshadowing biological factors, such as age, sex, or cancer stage. This variance was largely independent of biological characteristics and decreased in higher‐order components, suggesting that platelet‐specific signals remain robust and interpretable. These findings highlight the challenges posed by leukocyte‐associated RNA retention, while also affirming the resilience of platelet transcriptomic signatures for liquid biopsy applications. Notably, platelets may acquire RNA not only from circulating leukocytes but also directly from tumor‐associated inflammatory cells, further complicating the interpretation of transcriptomic signals attributed to leukocyte‐associated RNA retention [20].

RFR model identified measured technical factors—sequencing library size (feature importance: 0.38) and hospital‐specific protocols (0.33)—as the primary drivers of leukocyte‐associated RNA retention, whereas patient‐related variables, such as age (0.20) and cancer type (0.01), had minimal influence. The technical model achieved a high coefficient of determination (*R* ^2^ = 0.87), substantially outperforming the biological model (*R* ^2^ = 0.22), underscoring methodological variability as the predominant source of retention‐related variance. We cannot rule out that other yet unknown variables do also contribute to the variance of leukocyte‐associated RNA retention. Chebbo et al. [21] reported extensive transcriptomic alterations—affecting 8274 genes—resulting from leukocyte and erythrocyte retention in washed platelet samples, further emphasizing leukocyte‐associated RNA retention as a significant technical confounder. Similarly, analyses of breast cancer cohorts indicate that approximately 19% of transcriptomic variability is attributable to hospital‐specific protocols, including alterations in genes directly involved in platelet function. These findings highlight the critical importance of standardizing platelet isolation and processing methods across studies to ensure reproducibility and reliability in transcriptomic analyses [22], potentially using additional centrifugation, beads‐based or filter‐based approaches.

ML classifiers (including logistic regression, SVMs, random forests, and XGBoost) showed minimal performance differences when evaluated on high‐ versus low‐retention test sets. Logistic regression achieved AUC ROC values of 0.89 ± 0.02 (low retention) and 0.91 ± 0.01 (high retention), with statistically significant differences (*P* < 0.05) observed only for logistic regression, balanced random forests, and K‐nearest neighbors. When models were trained exclusively on either high‐ or low‐retention training sets, performance generally declined. Notably, models trained on high‐retention data performed better in several cases, including SVMs (AUC 0.84 vs. 0.76, *P* = 0.0003), LightGBM (0.79 vs. 0.74, *P* = 0.0006), and XGBoost (0.74 vs. 0.71, *P* = 0.0085). However, most models did not show significant differences (e.g., Logistic Regression, *P* = 0.5307). It should be noted here that these classifiers were trained on a different cohort as compared with the models created in the original datasets manuscript [8], hampering a direct head‐to‐head comparison of biosource performance. Overall, leukocyte‐derived signals contribute only marginal variability, reaffirming the diagnostic robustness of platelet RNA, potentially helping resolve some of retention‐related concerns previously raised by Banerjee et al. [16]. The key ML result is that leukocyte‐associated RNA retention is visible in expression space but has limited overlap with the cancer‐detection signal in the present analysis.

To reduce confounding from leukocyte‐associated RNA retention in existing datasets, we proposed two retrospective correction approaches—RUV‐based method and a linear regression–based method and we compared them to the RUV‐based method from In't Veld et al. [8]. Each correction was applied prior to DESeq normalization. Both RUV approaches substantially mitigated leukocyte‐driven variability, lowering the Calinski–Harabasz index from 661.90 to 12.20 and 5.78, respectively. However, classification performance on the low‐ and high‐retention test sets remained significantly different. For the leukocyte‐retention–based RUV method, AUC ROC values were 0.90 ± 0.02 and 0.92 ± 0.01 (*P* = 0.001), while for the library‐size–based RUV method, AUC ROC values were 0.85 ± 0.02 and 0.92 ± 0.01 (*P* = 5.588e‐9), respectively. Linear correction more moderately reduced the retention‐related signal (index reduced to 99.49), with no significant performance difference between retention levels (AUC ROC 0.88 ± 0.02 for both; *P* = 0.792). Compared with uncorrected data (Logistic Regression AUC ROC 0.89 ± 0.02 for low‐retention and 0.91 ± 0.01 for high‐retention samples), all methods maintained comparable classification performance. However, RUV's aggressive correction may eliminate biologically relevant signals, as previously cautioned by Chebbo et al. [21]. In contrast, linear correction offers a better balance between reducing technical bias and preserving diagnostic accuracy. It should be taken into account here that a clinically applied test, potentially based on platelet RNAs, has to be robust regarding technical (pre‐analytical) variables that influence the measured profiles, either by optimized sample processing technique and/or post hoc data correction methods. Because leukocyte‐associated transcripts may contain both technical noise and biologically relevant inflammatory information, correction strategies should be selected according to the specific analytical goal rather than applied indiscriminately.

This study is the first to evaluate the impact of leukocyte‐associated RNA retention on ML models using platelet bulk RNA‐sequencing data, to analyze such a large cohort of this scale (2351 samples), and to apply *post hoc* bioinformatic correction strategies. Chebbo et al. [21] identified 41 leukocyte‐associated genes in eight microarray samples, nine of which (PTPRC, CD3E, CD3D, CD53, RPS21, CD3G, RPL35, CCR7, and RPL27) overlap with our 138‐gene panel. These include canonical leukocyte markers (PTPRC, CD3E, CD3D, CD3G, CCR7, and CD53) and ribosomal genes (RPS21, RPL35, and RPL27). Like Chebbo et al., we observed a significant transcriptomic impact, but we attribute it to technical causes with minimal effect on cancer classification. Our previous work, Supernat et al. [6], established platelet transcriptomic stability in healthy donors across age and sex, identifying clusters associated with leukocyte genes (e.g., FCN1, ITGB2, LYZ, PTPRC, CTSS, and CPVL). We calculated that 22% of the healthy control samples had high‐leukocyte‐associated RNA retention (score > 33 100), which is similar to our current rate of 24%. Rondina et al. [23] also confirmed the platelet transcriptomic stability in healthy donors across different age and sex groups. Our findings align with these studies, as the variance in leukocyte‐associated RNA retention is orthogonal to age and sex, reinforcing its technical origin.

Our study has several limitations. First, while the 132‐gene leukocyte‐associated RNA retention score we developed is more comprehensive than single‐marker approaches, it remains an operational and compositional measure. It may therefore not capture all genes indicative of leukocyte‐associated RNA retention. This suggests that some leukocyte‐related transcripts may remain unaccounted for; however, the broader gene set reduces dependence on individual markers, such as PTPRC. In addition, CPM‐based scoring is not transcript‐length‐normalized, which should be considered when interpreting the score as a relative retention metric rather than an absolute measure of leukocyte RNA content. Second, the single‐cell RNA‐sequencing data used to define the leukocyte‐associated signature, particularly for platelet‐classified droplets, was limited in size and may not fully represent the heterogeneity of platelet transcriptomes. Only 91 of 913 platelet‐classified barcodes passed the stringent quality‐control threshold, and these droplets were co‐isolated during density‐gradient blood‐fraction processing rather than obtained from a platelet‐optimized isolation protocol. Therefore, they may not fully represent the circulating platelet population. Moreover, leukocyte expression patterns differed between samples from different institutions, including ovarian cancer and benign controls as well as healthy controls and breast cancer patients, which may introduce reference‐dependent effects. Nonetheless, this approach remains more comprehensive than single‐gene or purely bulk‐based definitions. Third, RNA‐based annotation cannot completely resolve the biological and technical sources of leukocyte‐associated signal in platelet RNA‐seq libraries. The score may capture residual leukocyte RNA, ambient RNA, platelet–leukocyte doublets, or platelet–leukocyte aggregates. Importantly, platelet–leukocyte aggregates are not only a technical concern but may also reflect biologically relevant platelet–immune interactions, especially in inflammatory and oncological settings. Therefore, the detected signal should be interpreted as leukocyte‐associated RNA retention rather than as a direct count of contaminating leukocytes. Fourth, the platelet pan‐cancer data were aligned to the GRCh37 reference genome, in line with most prior platelet transcriptomic studies in the context of liquid biopsy. In contrast, the single‐cell RNA‐seq data were aligned with GRCh38, the current standard for single‐cell workflows, which may introduce alignment‐related and annotation‐related discrepancies. To reduce this risk, we applied strict gene‐level harmonization, which led to the exclusion of six candidate genes from the final score. Fifth, unmeasured technical confounders, such as variations in sample storage or centrifugation, could influence leukocyte‐associated RNA retention beyond the identified drivers. Sixth, our primary matched low/high analysis deliberately focused on high‐ versus low‐retention samples to reduce clinical heterogeneity and create a strong contrast between retention groups. This design strengthens the assessment of retention‐associated transcriptomic effects but reduces direct comparability with larger, unstratified studies, such as Jopek et al. [18] and In't Veld et al. [8]. Seventh, although our classification pipeline is designed primarily for mRNA analysis (using SMARTer protocols), and thus does not incorporate long noncoding RNAs or circular RNAs, recent evidence suggests that platelet storage and handling may significantly influence noncoding RNA content [24]. Finally, the generalizability of our findings across diverse cancer types may be limited. Tumor‐specific platelet interactions could modulate the impact of leukocyte‐associated RNA retention, and further studies are needed to evaluate these effects in a cancer‐type‐specific manner. Rare tumor‐cell‐derived RNA also cannot be entirely excluded in individual samples, although it is unlikely to explain the cohort‐wide leukocyte‐associated retention gradient.

In this study, we aimed to answer the following research questions: (1) how can leukocyte‐associated RNA retention be reliably estimated *in silico*; (2) to what extent does leukocyte‐associated RNA retention introduce transcriptional noise into platelet RNA profiles; (3) is leukocyte‐associated RNA retention primarily driven by biological variability among patients or by technical factors during sample processing; (4) how does leukocyte‐associated RNA retention influence the performance of ML models trained on platelet transcriptomic data; and (5) can the effects of leukocyte‐associated RNA retention be mitigated retrospectively in already sequenced datasets?

Firstly, we designed a 138‐gene panel based on a single‐cell dataset, selecting genes with low expression in platelets and high expression in leukocytes. By summing the CPM‐normalized expression values of these genes, we were able to estimate leukocyte‐associated RNA retention levels in platelets pan‐cancer data. Secondly, we found that leukocyte‐associated RNA retention has a substantial impact on the platelet transcriptome. In fact, the resulting noise was more pronounced than that introduced by factors such as cancer stage, blood collection unit, or even information if a patient has cancer or not. Thirdly, using a RFR model, we demonstrated that leukocyte‐associated RNA retention is largely influenced by technical variability, particularly the blood collection unit and library size. Fourthly, we conducted a series of experiments to assess the impact of leukocyte‐associated RNA retention on ML models. Overall, performance was similar between the high‐ and low‐retention groups, with most differences not reaching statistical significance. However, we observed a slight but consistent improvement when training on the high‐retention group, which may offer benefits in cancer‐detection tasks. Lastly, we developed and applied two leukocyte reduction methods—RUV and linear correction—both of which significantly reduced leukocyte‐associated RNA retention, resulting in cleaner and more reliable transcriptomic data.

## 5 Conclusions

This study confirms that platelet RNA is the primary source of signals for cancer classification, while leukocyte‐associated RNA retention has only a modest effect on overall model performance. Nevertheless, transcripts derived from retained leukocytes may offer complementary information and could enhance classification accuracy in certain contexts. For biologically oriented investigations aimed at elucidating disease mechanisms, high sample purity remains essential, requiring stringent isolation methods (such as filtration or anti‐CD45 bead selection), quality control based on leukocyte‐associated RNA retention scores and possibly novel *in silico* correction methods limiting this effect. In contrast, diagnostic applications may benefit from cautious incorporation of leukocyte‐derived signals, a strategy that merits further evaluation. Moving forward, standardized protocols and carefully selected correction methods will be crucial in maintaining a balance between preserving informative signals and ensuring interpretability in platelet‐based liquid biopsy studies.

## Conflict of interest

A.S., M.S., K.P., J.J., A.J.Ż., M.T.R., and M.G.B are inventors on granted patents and pending patent applications related to the use of platelet transcriptomics for cancer detection.

## Author contributions

AS and MTR conceptualized the study. AS, KP, and MS designed the study. MS, KP, and ZC performed the data processing. MS, KP, and ZC performed the experiments and analysis. MS. and KP generated the graphics. SŁ‐S recruited the patients. SŁ‐S and TS provided the clinical data. AS‐C, PJ, and AS were involved in the single‐cell sequencing experiment execution. AS, MS, KP, MTR, SGJGIV, and MGB interpreted the results. MS visualized the data. MS, KP, and AS wrote the draft of the manuscript. AS supervised the study. AS, AJŻ, and JJ provided the funding, and reviewed and edited the manuscript. All authors approved the final version of the manuscript.

## Supporting information


**Fig. S1.** (A, B) UMAP visualizations of the single‐cell reference.
**Fig. S2.** (A, B) Comparison of highly expressed platelet‐associated genes between bulk and single‐cell reference data.
**Fig. S3.** Heatmap of genes used to estimate the level of leukocyte‐associated RNA retention in the single‐cell PBMCs dataset.
**Fig. S4.** Correlation between PTPRC‐only score and the final 132‐gene leukocyte‐associated RNA retention score.
**Fig. S5.** Correlation between final mapped 109‐gene nonribosomal sensitivity score and the final 132‐gene leukocyte‐associated RNA retention score.
**Fig. S6.** (A, B) Principal component analysis projections of the platelet pan‐cancer dataset, colored by leukocyte‐associated RNA retention scores, using principal components 2 through 4.
**Fig. S7.** Direct correlation between final 132‐gene leukocyte‐associated RNA retention score and library size.
**Fig. S8.** Principal component analysis 3D projections of the entire platelets pan‐cancer dataset, before and after RUV correction based on library size.
**Fig. S9.** Adjusted predicted probabilities vs. leukocyte‐associated RNA retention for PSO‐SVM predictions.
**Fig. S10.** ROC curves for PSO‐SVM probabilities—original and recalibrated using leukocyte score.
**Table S1.** Platelet‐classified droplet QC summary by sample.
**Table S2.** Markers used for cell‐type detection.
**Table S3.** Score definitions and gene‐count audit.
**Table S4.** Information about samples in the truncated dataset after matching.
**Table S5.** Optuna hyperparameter optimization overview and model‐specific search‐space summary.
**Table S6.** Names of markers used to calculate untargeted leukocyte‐associated RNA retention estimation.
**Table S7.** Names of negative control genes used for RUV correction based on leukocyte score.

## Data Availability

The single‐cell data are available at https://doi.org/10.5281/zenodo.17620564. Platelet RNA‐seq data is available at https://www.ncbi.nlm.nih.gov/geo/query/acc.cgi?acc=GSE183635. The code is available at the following GitHub link: https://github.com/melehean/Leukocyte_Retention.
