# Supplementary material for: The impact of leukocyte‐associated RNA retention on platelet RNA‐based cancer detection
Source: Mol Oncol. 2026 Oct 30:10.1002/1878-0261.70353. Online ahead of print. doi: 10.1002/1878-0261.70353 (PMC13722837; doi:10.1002/1878-0261.70353)
Supplement: Supplementary file 1 [file MOL2-9999-0-s001.docx]

*Supporting Information for*
**The impact of leukocyte-associated RNA retention on platelet RNA-based cancer detection
Authors:** Michał Sieczczyński, Krzysztof Pastuszak, Anna Samelak-Czajka, Zuzanna Cemka, Paulina Jackowiak^,^ Tomasz Stefaniak, Sylwia Łapińska-Szumczyk, Jacek Jassem, Anna J. Żaczek, Matthew T. Rondina^†^, Myron G. Best^†^, Sjors G.J.G. In 't Veld^†^, Anna Supernat*

*Corresponding author e-mail: anna.supernat@gumed.edu.pl

†These authors contributed equally.

**Table S1** Platelet-classified droplet QC summary by sample.

| **Sample** | **Platelets before QC** | **Platelets after QC** | **Pass rate (%)** |
| --- | --- | --- | --- |
| bctrl_1 | 18 | 3 | 16.67 |
| bctrl_2 | 5 | 0 | 0.00 |
| bctrl_3 | 38 | 6 | 15.79 |
| ctrl_1 | 19 | 2 | 10.53 |
| ctrl_2 | 13 | 0 | 0.00 |
| ctrl_4 | 12 | 5 | 41.67 |
| hgsoc_1 | 8 | 0 | 0.00 |
| hgsoc_2 | 166 | 15 | 9.04 |
| hgsoc_3 | 8 | 0 | 0.00 |
| hgsoc_4 | 0 | 0 | NA |
| hgsoc_5 | 20 | 2 | 10.00 |
| hgsoc_6 | 28 | 0 | 0.00 |
| hgsoc_7 | 28 | 4 | 14.29 |
| hgsoc_8 | 98 | 13 | 13.27 |
| hgsoc_9 | 22 | 3 | 13.64 |
| hgsoc_10 | 25 | 3 | 12.00 |
| hgsoc_11 | 16 | 2 | 12.50 |
| hgsoc_12 | 70 | 3 | 4.29 |
| hgsoc_13 | 9 | 0 | 0.00 |
| brca_1 | 24 | 5 | 20.83 |
| brca_2 | 12 | 4 | 33.33 |
| brca_3 | 16 | 2 | 12.50 |
| brca_4 | 74 | 6 | 8.11 |
| brca_5 | 24 | 4 | 16.67 |
| brca_6 | 14 | 0 | 0.00 |
| brca_7 | 140 | 9 | 6.43 |
| brca_8 | 4 | 0 | 0.00 |
| brca_9 | 2 | 0 | 0.00 |
| brca_10 | 0 | 0 | NA |
| **Pooled total** | **913** | **91** | **10.0** |

**Figure S1** UMAP visualizations of the single-cell reference. **(A)** UMAP plot colored by sample name, used to assess the relationship between cell-type structure and sample as well as cohort origin. **(B)** UMAP plot colored by cell-type annotation, which was used to define the single-cell reference for constructing leukocyte-associated RNA signals.

**Table S2** Markers used for cell-type detection.

| **UMAP category** | **Markers used for detection** |
| --- | --- |
| B cells | MS4A1, CD19 |
| CD4 T cells | CD4, IL7R |
| CD8 T cells | CD8A, CD8B |
| Regulatory T cells | IL2RA, FOXP3, TIGIT |
| NK cells | KLRF1, NCAM1 |
| Macrophages | CD68 |
| Monocytes | FCN1, LYZ, FCGR3B, S100A9 |
| Neutrophils | FUT4, CEACAM8, ITGAM, ITGB2 |
| Dendritic cells | FCER1A, CD1C, AXL, CD83 |
| Fibroblasts | PDGFRB, FAP |
| Platelets | PPBP, ITGB3, PF4, GP9, ITGA2B |

**Figure S2** Comparison of highly expressed platelet-associated genes between bulk and single-cell reference data. **(A)** Top expressed genes in bulk platelet RNA-seq samples. **(B)** Expression of the same top bulk platelet genes in platelet-classified pseudobulk profiles derived from the single-cell reference.

**Table S3** Score definitions and gene-count audit.

| **Score or subset** | **Gene count** | **Definition / counting rule** | **Use in revised analyses** |
| --- | --- | --- | --- |
| Candidate leukocyte-associated discovery panel | 138 | Genes selected from the single-cell blood-fraction reference under the original zero-expression criterion and recovered under the relaxed <1% platelet-expression sensitivity criterion. | Marker-discovery panel. |
| Final bulk leukocyte-associated RNA retention score | 132 | 138 candidate genes after strict cross-resource gene-level harmonization; NOP53, ATP5MC2, RSRP1, TRBC1, ATP5MG, and SARAF were excluded. | Primary operational score used for bulk analyses. |
| Ribosomal-protein genes in the discovery panel | 23 | 12 RPL* genes plus 11 RPS* genes. | Used to define the non-ribosomal sensitivity subset. |
| Non-ribosomal candidate subset before strict harmonization | 115 | 138 candidate genes minus 23 ribosomal-protein genes. | Pre-harmonization subset for the non-ribosomal sensitivity analysis. |
| Final mapped non-ribosomal sensitivity score | 109 | 115 non-ribosomal candidates minus the same six unmappable genes excluded by strict harmonization. | Sensitivity score used to address ribosomal-gene concerns. |
| PTPRC-only score | 1 | PTPRC/CD45 only. | Single-gene leukocyte-associated proxy that avoids multi-gene transcript-length weighting. |

**Table S4** Information about samples in the truncated dataset after matching – the number of samples according to sex, age, blood collecting unit, information on population (cancer patients or asymptomatic controls), cancer stage, and cancer type, as well as median and IQR (interquartile range) of total leukocyte-associated RNA retention.

|  | **All Samples** | **Low Leukocyte Group** | **High leukocyte Group** |
| --- | --- | --- | --- |
| **Number of samples** | 754 | 377 | 377 |
| Median leukocyte-associated RNA retention score (IQR) | 22400.80 (41515.89) | 6754.40 (3365.02) | 48283.40 (15968.27) |
| Sex | | | |
| Male | 395 | 204 | 191 |
| Female | 359 | 173 | 186 |
| Age | | | |
| <30 | 35 | 16 | 19 |
| 30-40 | 35 | 18 | 17 |
| 40-50 | 91 | 54 | 37 |
| 50-60 | 199 | 103 | 96 |
| 60-70 | 242 | 118 | 124 |
| 70-80 | 127 | 57 | 70 |
| >80 | 25 | 11 | 14 |
| Blood collecting unit | | | |
| Institute 1 | 1 | 0 | 1 |
| Institute 2 | **22** | 13 | 9 |
| Institute 3 | 10 | 9 | 1 |
| Institute 4 | 13 | 11 | 2 |
| Institute 5 | 184 | 20 | 164 |
| Institute 6 | 5 | 1 | 4 |
| Institute 7 | 5 | 1 | 4 |
| Institute 8 | 40 | 18 | 22 |
| Institute 9 | 52 | 18 | 34 |
| Institute 10 | 17 | 14 | 3 |
| Institute 11 | 13 | 7 | 6 |
| Institute 12 | 2 | 1 | 1 |
| Institute 13 | 390 | 264 | 126 |
| Population | | | |
| Cancer patients | 578 | 289 | 289 |
| Asymptomatic controls | 176 | 88 | 88 |
| Cancer stage | | | |
| Stage I | 29 | 12 | 17 |
| Stage II | 33 | 14 | 19 |
| Stage III | 78 | 39 | 39 |
| Stage IV | 357 | 183 | 174 |
| Unknown stage | 81 | 41 | 40 |
| Asymptomatic controls | 176 | 88 | 88 |
| Cancer type | | | |
| Non-small-cell lung cancer | 318 | 159 | 159 |
| Head and neck cancer | 40 | 20 | 20 |
| Pancreatic cancer | 38 | 19 | 19 |
| Glioma | 38 | 19 | 19 |
| Ovarian cancer | 28 | 14 | 14 |
| Prostate cancer | 26 | 13 | 13 |
| Melanoma | 18 | 9 | 9 |
| Sarcoma | 14 | 7 | 7 |
| Urothelial cancer | 12 | 6 | 6 |
| Multiple Myeloma | 10 | 5 | 5 |
| Colorectal cancer | 10 | 5 | 5 |
| Endometrial cancer | 10 | 5 | 5 |
| Cholangiocarcinoma | 6 | 3 | 3 |
| Esophageal carcinoma | 6 | **3** | **3** |
| Lymphoma | 4 | 2 | 2 |

**Table S5** Optuna hyperparameter optimization overview and model-specific search-space summary. A separate Optuna study was run for each classifier. Each trial trained on the training set and optimized validation ROC AUC from predicted probabilities. Test ROC AUC was recorded but not used for hyperparameter selection.

| **Classifier** | **Hyperparameter families evaluated** |
| --- | --- |
| Elastic-net logistic regression | Regularization strength, elastic-net mixing parameter, and intercept fitting. |
| Support vector machine | Regularization parameter, kernel type, polynomial degree for polynomial kernel, and gamma for radial-basis-function kernel. |
| K-nearest neighbors | Number of neighbors, weighting strategy, and distance metric. |
| Random forest, balanced random forest, and extra trees | Number of trees, splitting criterion, minimum samples for node splitting, minimum samples per leaf, minimum impurity decrease, maximum depth, and bootstrap sampling. |
| AdaBoost | Number of estimators and learning rate. |
| Gradient boosting | Number of estimators, learning rate, minimum samples for node splitting, minimum samples per leaf, minimum impurity decrease, and maximum depth. |
| LightGBM | Number of leaves, learning rate, number of estimators, maximum depth, L1 and L2 regularization, minimum samples per child node, and class-imbalance handling. |
| XGBoost | Maximum depth, learning rate, number of estimators, L1 and L2 regularization, and minimum child weight. |

**Table S6** Names of markers used to calculate untargeted leukocyte-associated RNA retention.

IL7R, PTPRC, CD3E, CD52, CD3D, CD53, NOP53, NCL, NPM1, CD37, CD44, CNBP, COMMD6, EEF1D, HSP90AB1, KLF2, RPL10A, SLC25A6, TMEM123, ATM, ATP5MC2, EEF1B2, HINT1, HNRNPDL, IL32, LEF1, LTB, PCED1B-AS1, PNISR, RPS21, TCF7, TRAC, TRAF3IP3, ZFP36L2, CYBA, EEF2, FAM107B, GIMAP7, LDHB, RPL22, RPL38, RPLP0, S100A10, S100A6, SAMHD1, SELL, ANAPC16, ARGLU1, BTG1, CD3G, CD48, CD74, CD96, CLEC2D, CORO1A, COX7C, CYTH1, EIF3E, ETS1, EVL, FOXP1, HSPA8, LINC00861, LUC7L3, RPL35, RPS5, RSRP1, SNHG5, SNRPD2, SRRM2, STK17B, TRBC1, TRBC2, BACH2, CD2, CD69, EIF3K, FNBP1, FUS, GAS5, IKZF1, LAPTM5, LCP1, LEPROTL1, PDE3B, PFDN5, PRRC2C, RBL2, RPL29, RPS26, RPS8, RPS9, S100A4, SLC38A1, SMAP2, SRSF11, STAT4, TOMM7, ZFAS1, ANKRD44, ATP5MG, BCL11B, BCL2, BTBD9, CAMK4, CCR7, EIF4A2, EML4, FKBP5, HNRNPA3, HNRNPU, HP1BP3, ITK, LCK, MAML2, OXNAD1, PIK3IP1, PPIA, RCAN3, RPL12, RPL24, RPL27, RPL36, RPL37A, RPL6, RPS11, RPS13, RPS23, RPS25, RPS4X, RPSA, SARAF, SKAP1, SON, SORL1, SP100, SRSF7, YWHAB

**Figure S3** Heatmap of the 138 candidate leukocyte-associated genes identified from the single-cell reference. Columns represent pseudobulk samples of patient leukocytes (N=29) or platelet-classified droplets (N=18), with values shown as z-scores of counts-per-million (CPM)-normalized aggregated raw counts. The candidate panel was used for marker discovery; after strict cross-resource gene-level harmonization, 132 reliably mappable genes were retained for the final bulk leukocyte-associated RNA retention score. Platelets were found only in 18 samples. Notably, two distinct leukocyte clusters emerged: one consisting of ovarian cancer samples and benign gynecologic controls, and another comprising breast cancer samples and healthy controls. Despite being generated independently using different library preparation technologies, both datasets show a clear and reproducible separation between platelet and leukocyte expression profiles—regardless of the library construction method—demonstrating that the platelet transcriptome is fundamentally distinct from that of leukocytes. Notably, the leukocyte profiles also exhibit greater heterogeneity compared to the relatively uniform expression patterns observed in platelets. However, using the compiled leukocyte gene panel, some platelet samples express these leukocyte markers, possibly due to co-expression of such markers or potential co-isolation of single platelets with leukocytes.

**Figure S4** Correlation of PTPRC-only score and the final 132-gene leukocyte-associated RNA retention score (Pearson r = 0.831; Spearman ρ = 0.913; both p < 0.001).

**Figure S5** Correlation between final mapped 109-gene non-ribosomal sensitivity score and the final 132-gene leukocyte-associated RNA retention score (Pearson r = 0.967; Spearman ρ = 0.990; both p < 0.001). The final mapped non-ribosomal sensitivity score contains 109 genes after applying strict harmonization to the analysis-defined 115-gene non-ribosomal candidate subset.

**Figure S6A-B.** Principal component analysis (PCA) projections of the platelet pan-cancer dataset, colored by leukocyte-associated RNA retention scores. In panels S6A and S6B, alternative principal components (PC2 vs. PC3 in S2A, PC3 vs. PC4 in S2B) are shown to illustrate the diminishing influence of leukocyte-related signal.

**Figure S7** Direct correlation between final 132-gene leukocyte-associated RNA retention score and library size (Pearson r = -0.362; Spearman ρ = -0.479; both p < 0.001).

**Table S7** Names of negative control genes used for RUV correction based on leukocyte-associated RNA retention.

RBM6, ST7, SKAP2, PRKAR2B, CD9, GABARAPL2, RAB27B, ARAP2, MCUR1, SYNE2, USP36, GUCY1B3, SFSWAP, MYLK, MSANTD3, KIF2A, RDH11, ACAP1, ARHGEF1, PCNP, CDC14B, CTTN, DYNLL1, RPL6, NLRP1, SNAP23, POLRMT, TPTEP1, GRAP2, ZFYVE21, PABPC1L, TUBB1, PGRMC1, TSC22D1, PIEZO1, RAB11A, KCNN4, SNRNP70, FBL, NKG7, DNAJB6, SPOCK2, ANKRD26, KAT2A, YWHAE, MMD, CEP164, CDK2AP1, ENO2, ACVR1, GRB14, SSX2IP, PRDX6, MFSD1, CCDC92, CTNNAL1, LGALSL, PAIP2, RNF11, ARHGAP9, LRP1, VAPB, VAMP7, F13A1, WRNIP1, DYNLRB1, ST3GAL3, TUBA4A, GNG11, TUBGCP6, GNAZ, NDUFA5, CDKN2D, SAT1, SH3BGRL, PRR7, PNISR, ZRANB2, WASF3, ACTR3B, C12orf39, SP110, ITM2B, IGF2BP3, IL11RA, SLC22A23, SORL1, PLCB2, RPS24, ABI2, PSTPIP1, MAP1LC3B, TMC6, ATHL1, COL6A2, SYTL1, PRUNE, AIG1, MEPCE, TMEM140, C9orf142, RSU1, PARD3, RGS10, CD226, PIP4K2A, ASAP2, CCDC175, PSTPIP2, MBNL1, DAB2, BMP6, ASAP1, ANGPT1, TMEM55A, TTN, GNAQ, C15orf26, NPTN, SPPL3, TSPAN33, DEDD, RLTPR, CD3G, CMPK1, NEXN, IGSF8, CCDC141, SLMAP, CXCL3, PF4, MTHFD2L, ELOVL7, YWHAZ, CRYL1, DPCD, TP53I13, PPP1R14A, TMC8, RPSA, LTBP3, PCMTD1, RAB31, SDPR, LRRC28, HEXDC, LIMS1, REPS2, UBE2E3, CAMTA1, NADSYN1, MIR4435-1HG, MSRB3, ANKRD36C, GOLGA8A, LSM1, MCTP1, RUFY1, BEND2, CD19, DMAP1, MTURN, HIST1H2AC, HIGD1A, RGS6, EFHC2, TMEM50A, SNN, SLC24A3, ANO9, PRKG1, CIDECP, SESTD1, EPOR, TRPV2, PTPLAD2, HMGB1, MPHOSPH8, ANKRD36B, GZMM, HIST1H2BK, DNM3, MAN1A2, SH3BGRL2, C1orf228, TLK1, CAPZA2, ARMCX6, EFCAB2, CXorf65, SMIM5, TMSB4X, LEPROT, CD27-AS1, TNFRSF25, CCNL2, SLC26A6, LTB, OST4, STXBP5-AS1, AMY2B, OIP5-AS1, MALAT1, ZNF271, MYO15B, CASC15

**Figure S8** Principal component analysis 3D projections of the entire platelets pancancer dataset, before and after correction, grouped by leukocyte-associated RNA retention levels (low, medium, and high). Raw data was normalized with and without RUV-correction based on library size.

**Figure S9** Adjusted predicted probabilities vs. leukocyte-associated RNA retention (center-adjusted; library size at mean). Shaded bands are 95% CIs. Dashed vertical lines mark the 10th/50th/90th percentiles. Curves show strong separation across the data-dense range; the shape indicates a retention-related calibration shift, not a collapse of discrimination. In addition, we modeled the published probability itself (quasi-binomial logit) and tested whether leukocyte-associated RNA retention score, sequencing depth, and center explain additional variation beyond the biological group label. Adding the leukocyte-associated RNA retention score (cubic spline) improved the fit (ΔDev = 11.39 on 3 df, F = 11.64, p = 1.6×10⁻⁷). Adding library size improved the fit (ΔDev = 2.70 on 1 df, F = 8.30, p = 0.0040). Adding the inclusion center resulted in a substantial improvement (ΔDev = 14.68 on 6 df, F = 7.79, p = 3.1×10⁻⁸). We conclude that the published probabilities are systematically shifted by leukocyte-associated RNA retention and center, and to a lesser extent by sequencing depth—i.e., a calibration issue while biological separation remains. In this analysis, we obtained the original sample set and made a prespecified selection. We included only malignant and healthy-donor (non-malignant) samples, removed records with missing age and/or gender, and restricted to centers with >20 total samples. After matching leukocyte-associated RNA retention scores, the final analysis set contained N = 1,167 samples from 7 centers (GDANSK 27, MAAS 45, MGH 230, NKI 163, RAD 32, UMCG 113, VUMC 557). The final dataset contained 1,081 malignant and 86 non-malignant samples. The AUC of this sample set based on the published pan-cancer score was 0.911.

**Figure S10** ROC curves of the published pan-cancer score and the recalibrated pan-cancer score. We recalibrated the probabilities on the logit scale by adding the leukocyte-associated RNA retention score, library size, and center. We observed an improvement of AUC from 0.911 to 0.9535. The Brier score decreased from 0.0826 to 0.0471 (≈ 43% relative reduction). The calibration intercept/slope went from 1.381 / 0.988 (global underestimation) to 0.021 / 0.975 (near-ideal). Thus, recalibration corrects the systematic shifts and improves both ranking and absolute probability accuracy (noting that these are apparent, in-sample metrics).
